# *DeepScreen*: An Accurate, Rapid, and Anti‐Interference Screening Approach for Nanoformulated Medication by Deep Learning

**DOI:** 10.1002/advs.201800909

**Published:** 2018-07-23

**Authors:** Yanjing Zhu, Ruiqi Huang, Rui Zhu, Wei Xu, Rongrong Zhu, Liming Cheng

**Affiliations:** ^1^ Division of Spine Surgery Department of Orthopedics Tongji Hospital Tongji University School of Medicine Shanghai 200065 China; ^2^ Key Laboratory of Spine and Spinal Cord Injury Repair and Regeneration Tongji University Ministry of Education Shanghai 200065 China; ^3^ Clinical Research Center for Brain and Spinal Cord Tongji University Shanghai 200065 China

**Keywords:** anti‐interference, convolutional neural networks, deep learning, drug screening, nanocarriers

## Abstract

Accuracy of current efficacy judgment methods for nanoformulated drug remains unstable due to the interference of nanocarriers. Herein, *DeepScreen*, a drug screening system utilizing convolutional neural network based on flow cytomerty single‐cell images, is introduced. Compared to existing experimental approaches, the high‐throughput system has superior precision, rapidity, and anti‐interference, and is cost‐cutting with high accuracy. First, it can resist most disturbances from manual factors of complicated evaluation progress. In addition, class activation maps generated from *DeepScreen* indicate that it may identify and locate the tiny variation from cell apoptosis and slight changes of cellular period caused by drug or even nanoformulated drug action at very early stages. More importantly, the excellent performance of assessment on two types of nanoformulations and fluorescent drug proves the fine generality and anti‐interference of this novel system. All these privileged performances make *DeepScreen* a very smart and promising system for drug detection.

## 1 Introduction

Nanomaterials have been a hotspot in novel drug research recently, which have been reported of high potency to improve the native drug efficacy.^1^, ^2^ However, existing approaches for evaluating the nanoformulated drugs are mainly based on traditional medication. As to nanoformulated drug screening, additional challenges arise from the special physicochemical properties of nanoparticles.^3^, ^4^, ^5^ Nanoparticles might distort the most of in vitro classical cytotoxicity assay including 3‐(4,5‐dimethylthiazol‐2‐yl)‐2,5‐diphenyltetrazolium bromide (MTT) and lactate dehydrogenase release assays. The interference could be caused by several reasons: the intrinsic high absorbance, optical activity, and their large surface area and high energy.^6^, ^7^, ^8^ The common methods can no longer meet the requirements for novel nanoformulated drug discovery, and the emergence of a new method is urgently needed.

An ideal screening system requires fine generality and ability to adapt to growing innovation of nanoformulated drug development. We notice that machine learning has been extensively used in medical research and gain some progress.^9^, ^10^ Recent researches demonstrate that machine learning could identify some indiscoverable change of cells and predict their variation tendency or process.^11^, ^12^ Machine learning approaches could distinguish the slight change of treated cells in the earlier stage of drug function and realizing a self‐acting evaluation without much intervention from subjective action. We also notice that machine learning‐based virtual drug screening systems have increasingly emerged recently, providing a theoretical framework for predicting drug properties and compound structure prioritization.^13^, ^14^, ^15^ But traditional structure and ligand‐based machine learning approaches are highly limited by the obtainable database and manually classified features, which restrict their promotion and application. Therefore, we consider building an approach based on images to bypass those limitations of recent judgment methods for nanoformulated drugs.

To fully exploit the information of the abundant image patches, convolutional neural network (CNN) has been chosen for the drug screening system. Compared to traditional machine learning methods, convolutional neural network is designed to tackle regression and classification tasks based on image‐like data. A typical convolutional neural network usually contains two types of layers, namely, convolution layer and pooling layer. These layers are capable of extracting features from previous layers with ease. Stacking many of these layers together, we can form a convolutional neural network to automatically extract features from input single‐cell image and then perform classification.^16^, ^17^, ^18^

**Figure 1:**
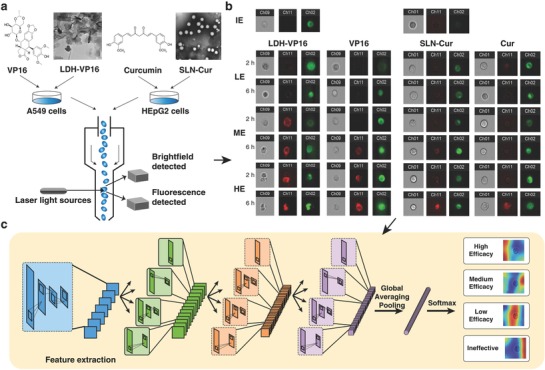
An overview of the *DeepScreen* workflow. a) A549 and HEpG2 cells were treated with several concentrations of VP16/LDH‐VP16 and Cur/SLN‐Cur, separately. TEM photos of LDH‐VP16 and SLN‐Cur were shown, and the scale bar was 100 nm and 0.2 µm, separately. Then after 2 and 6 h, cells were stained with Annexin V‐APC, collected and managed by image flow cytometry. A549 cells were stained by anti‐EGFR‐FITC after Annexin V progress. b) Bright‐field and dark‐field images (Annexin V‐APC channel shows red, while anti‐EGFR‐FITC was green) were obtained, respectively. For HEpG2 cells, the green channel presents the fluorescence interference of Curcumin. IE, LE, ME, and HE present ineffective, low, medium, and high efficacy, respectively. c) Gained images were used as training data for the screening system, a schematic of the convolutional neural network (CNN) was demonstrated. The procedure of class activation mapping (CAM) technique was also shown.

Therefore, as shown in **Figure** **1**, *DeepScreen*, a novel deep learning‐based drug screening system, is designed and developed here. Two kinds of nanoformulated drug system, inorganic‐layered double hydroxide loaded with etoposide (LDH‐VP16) and lipid‐based materials solid lipid nanoparticles loaded with Curcumin (SLN‐Cur), were chosen as test examples for our novel screening system.^19^, ^20^ Millions of drug‐treated single‐cell images were obtained from flow cytometry and used to build a classifier that evaluates the efficacy of the drug.

Over here we introduce the detailed development of *DeepScreen* system, and prove its fine properties of precise, fast, low‐cost, and easy‐practice for testing nanoformulated drugs. Implemented in this manner, only simple operation of treating and collecting cells is demanded, no manual analysis is needed, which could greatly reduce the manual error. Besides, by virtue of deep learning, nuances caused by very short time treated could be clearly distinguished which effectively accelerate the test progress.

## 2 Results

### 2.1 *DeepScreen* System Is Established Using Single‐Cell Images Gained from Flow Cytometry

As shown in Figure S1a (Supporting Information), LDH, LDH‐VP16, SLN, and SLH‐Cur were well prepared for the following study, the Fourier transform infrared spectroscopy (FTIR) spectral showed the following selected bands: 3356 cm^−1^ (O—H groups), 1610 cm^−1^ (C=O stretch of carboxyl methyl), and 1485 cm^−1^ (C=C stretching in the backbone of the aromatic phenyl ring). The spectra of LDH showed characteristic peaks: 3526 cm^−1^ (O—H groups) and 1384 cm^−1^ (NO_3_^−^ stretching vibration).^21^ LDH‐VP16 showed 1610 cm^−1^ (C=O stretch of carboxyl methyl) and 1485 cm^−1^ (C=C stretching in the backbone of the aromatic phenyl ring) similar to VP16 and 3526 cm^−1^ (O—H groups) and 1384 cm^−1^ (NO_3_^−^ stretching vibration) similar to LDH. The anionic exchange reaction leading to the weaker band at 1384 cm^−1^ (NO_3_^−^ stretching vibration) indicates that VP16 was entrapped by the LDH. Figure S1b (Supporting Information) shows the FTIR results of Cur, SLN, and SLN‐Cur. Cur showed characteristic peaks at 1628 cm^−1^ (C=O stretch of carboxyl methyl) and 1430 cm^−1^ (—CH_3_ stretching of methyl groups). SLN exhibited the following characteristic absorption bands: 2917 cm^−1^ (C—H stretching vibration), 2849 cm^−1^ (C—H stretching vibration), and 1430 cm^−1^ (—CH_3_ stretching of methyl groups). SLN‐Cur showed both peaks of Cur and SLN demonstrate that Cur was well encapsulated by SLN.^22^ In accord with FTIR results, as shown in Figure S2 (Supporting Information), dynamic light scattering (DLS) shows that size of LDH‐VP16 (141.1 ± 1.7 nm) and SLN‐Cur (150.6 ± 1.9 nm) has a slight increase than LDH (98.96 ± 0.9 nm) and SLN (103.2 ± 1.5 nm), respectively.

**Figure 2:**
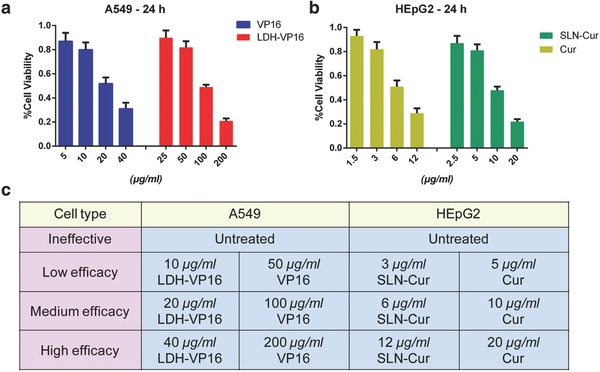
Benchmark was set up using certain efficacy agents treated cells. a) Viability of A549 cells after coincubated with VP16 or LDH‐VP16 in different concentrations for 24 h, respectively. b) Viability of HEpG2 cells after coincubated with Cur or SLN‐Cur in different concentrations for 24 h, respectively. c) Classification was set according to the cell viability, training data were divided into four types as ineffective, low, medium, and high efficacy.

MTT assay was applied to access the viability of 24 h‐treated A549 and HEpG2 cells as shown in **Figure** **2**a,b. We obtain the treated drug and the associated nanocarrier drug concentrations in accordance with 80, 50, and 20% cell viability, respectively, which demonstrate low, medium, and high efficacy of drug effect.

After that, A549 and HEpG2 cells were treated with concentrations above. However, this time, the incubation time was shortened from 24 h to 2 and 6h in order to reduce the period of drug screening process. We collected 103 568 and 115 968 single‐cell IFC images from treated and untreated A549 and HEPG2 cells separately as training data. Likewise, 25 892 A549 and 28 992 HEPG2 cell IFC images from independent experiments were generated as testing data. Each single‐cell IFC image consists of two separate channels, namely, bright‐field and Annexin V‐APC channel. As shown in Figure 2c, we label these IFC image data with ineffective, low, medium, and high efficacy (presented in Figure as IE, LE, ME, and HE) based on the treated drug and the associated nanocarrier drug concentrations from the 24 h experiment above.

Using the labeled IFC image data, the *DeepScreen* system is established based on convolutional neural network as Figure 1. This convolutional neural network contains 46 layers which should have sufficient depth and capability to extract high‐order features from the IFC image data. The model was trained on two NVIDIA GTX 1080Ti GPUs.

### 2.2 Deep Learning Provides a More Rapid, Accurate, and Convenient Approach for Screening Drug and Nanocarrier Drug System

**Figure 3:**
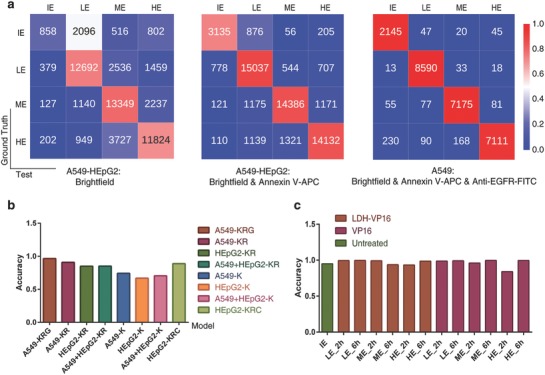
Deep learning provides accurate and rapid approach for screening drug and nanocarrier drug system. a) Confusion matrices for deep learning for classification of four classes. IE, LE, ME, and HE present ineffective, low, medium, and high efficacy, respectively. Color bar presents the accuracy. b) Accuracy of each test cell lines in a different set of models. *X*‐axis presents chosen training data of each model and color bars present the test data. K, R, G, and C present bright‐field, Annexin V‐APC, anti‐EGFR‐FITC, and self‐fluorescence of Cur channel, separately. c) Accuracy of LDH‐VP16, VP16, and untreated group in A549‐KRG model at 2 h/6 h under different stages of efficacy. IE, LE, ME, and HE present ineffective, low, medium, and high efficacy, respectively.

An excellent performance of the *DeepScreen* model is shown as list in **Figure** **3**a. In the case of using bright‐field and Annexin V‐APC channel images as training data, our model reached the high accuracy of 0.851, 0.864, and 0.908 in testing mixed cells, HEpG2, and A549, separately. As shown in Figure 3b and Table S1 (Supporting Information), no significant difference was observed in a single‐cell type model and the mixed one, which indicate that our model could distinguish different cell lines. The testing data were collected from 2 and 6 h treated cells, the notable accuracy shown in Figure 3c and Tables S2–S4 (Supporting Information) proved that compared with existing methods, the measuring time greatly reduced from couple of days to several hours, thus we present a more rapid approach for assessing treated efficacy. Besides, as the discussion above, we could hardly see the difference in treated cells by MTT and flow cytometry, which implied that our model performed better in evaluating the effect of drug and nanocarrier drug system at the early stage of function progress. Moreover, we also provide flexible options according to the users' requirement, for precise priority, an extra labeled marker such as anti‐EGFG‐FITC channel could be added to improve the accuracy to 0.966 (Tables S1 and S3, Supporting Information), while for convenience priority, Annexin‐V‐APC channel could be removed for a simpler progress, the accuracy has an acceptable slight reduction to 0.705 (Tables S1 and S4, Supporting Information) when using only bright‐field images.

**Figure 4:**
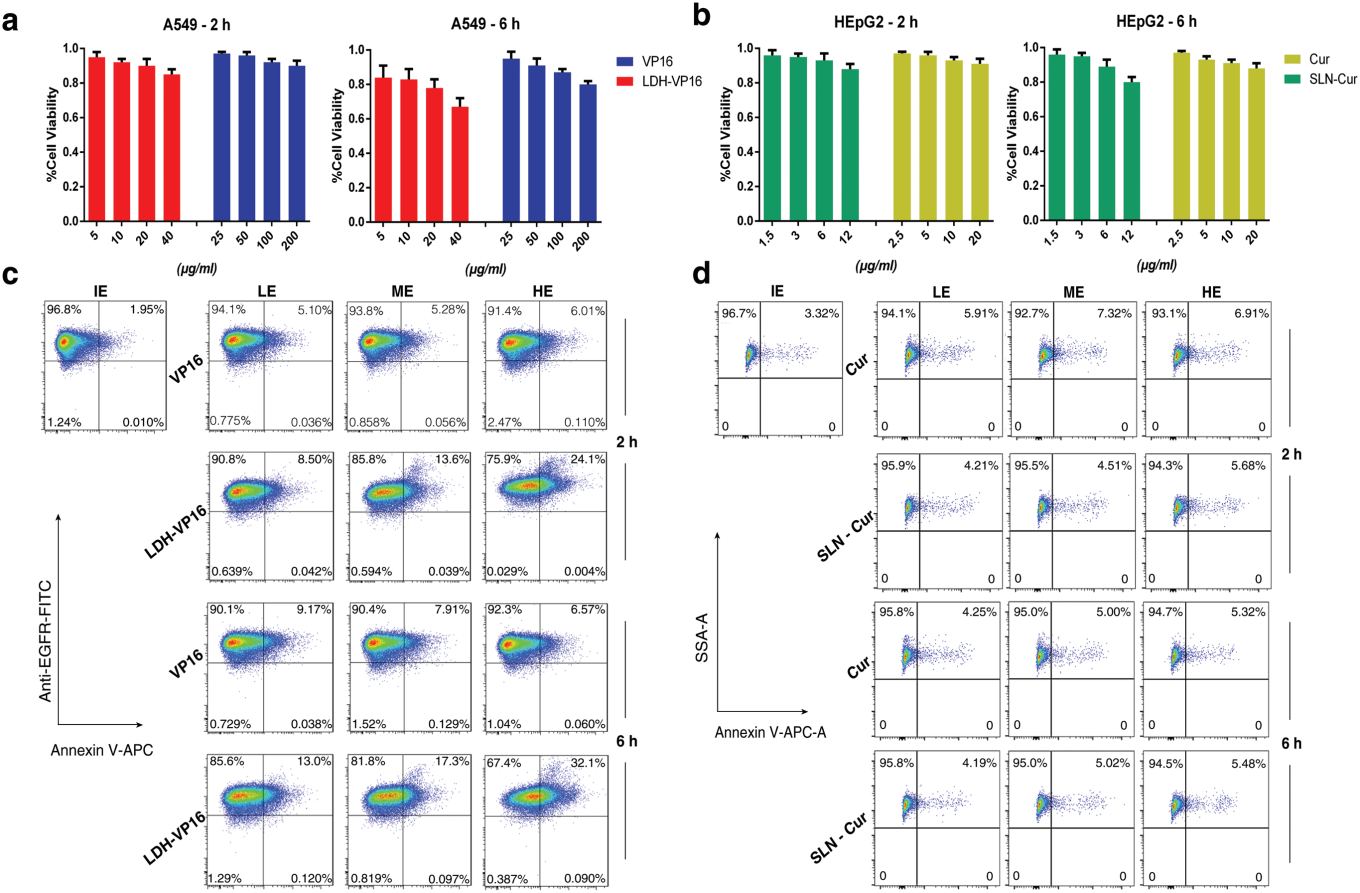
Existing approaches are lack of efficiency for nanoformulated drug screening. a,b) MTT assay failure to identify the different functional level of drugs after a short time treated. c) Annexin V‐APC/anti‐EGFR‐FITC flow cytometry analysis for VP16/LDH‐VP16 treated A549 cells for 2 and 6 h at various effective concentrations which are proven by 24 h MTT assays above. d) Annexin V‐APC flow cytometry analysis of Cur/SLN‐Cur treated HEpG2 cells at certain concentrations for 2 and 6 h. No significant difference could be observed in both the experiments. IE, LE, ME, and HE present ineffective, low, medium, and high efficacy, respectively.

Currently, common methods for evaluating the drug efficacy in the laboratory mainly include MTT assay and flow cytometry, which were all tested here for comparisons. As shown in **Figure** **4**a,b, by using MTT assay, no significant difference could be observed neither in VP16‐treated A549 cells nor Cur‐treated HEpG2 cells for 2 and 6 h, mainly because the absorbance‐based assay is not sensitive at picking out minor changes under short‐time treatment. As shown in Figure 4c,d and Figure S4 (Supporting Information), still no statistically significant difference could be observed by flow cytometry with a short treatment time with same staining.

### 2.3 Deep Learning Screening System Shows the Ability of Anti‐Interference

As discussed above, all the screening methods meet the interference of test agents' self‐fluorescence and other physicochemical properties. To estimate the generality of our approach, fluorescent drug Curcumin and typical nanocarriers were introduced to the modeling progress. Figure 3b and Table S1 (Supporting Information) demonstrated that the accuracy has no significant difference when the self‐fluorescence channel was added to consideration. Furthermore, we chose the well‐proved LDH‐VP16 and SLN‐Cur in our lab to test if our method was capable to function without influence.^23^, ^24^ LDHs are classical inorganic functional materials, the LDH‐VP16 delivery system displays a scroll shape, while SLNs are nanoparticles mainly formed by solid lipids and the drug carrier system generally shows spherical shape as transmission electron microscopy (TEM) images shown in Figure 1a.^7^, ^24^ Recent studies reveal that the morphology and composition have an impact on the action of nanomaterials, the complex interactions may confuse the evaluation progress setting of experimenters.^25^ Notably, the stable accuracy of testing nanocarrier drug delivery systems as shown in Figure 3b indicated that the *DeepScreen* model arrives fairly reliable in handling the complicated interference.

### 2.4 Class Activation Maps Offer an Insight into Medication Function Process

**Figure 5:**
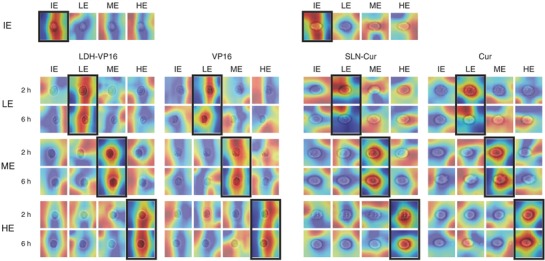
CAM highlights the class‐specific discriminative regions of cells. IE, LE, ME, and HE present ineffective, low, medium, and high efficacy, respectively.

Given an input image, a convolutional neural network will first extract high‐order features from it and then perform classification.^26^, ^27^ Moreover, the classification process is basically a linear combination of the extracted features from the previous layer of the classification layer.^28^, ^29^ To further understand the representation of our trained model, we introduce a model‐based visualization technique named class activation mapping as shown in **Figure** **5**. These class activation maps show how our model sees an input single‐cell image. When providing a correct prediction, the model tends to be concentrated on some specific area of the cell. In most cases, it focused on the border of the cell.

## 3 Discussion

The frequently used analytical flow cytometry machine is laser‐ or impedance‐based, the gained automated quantification of specified optical parameters on a cell‐by‐cell basis usually needs to be adjusted parameters like voltage and compensation.^30^, ^31^ The analysis of cell state relies on the common software, which is not able to detect the partial changing of each single cell and leading to the underutilization of the high‐throughput data. Here, we present *DeepScreen* as an efficient and effective computational model for practical drug screening to overcome the limitation of regular flow cytometry and make the utmost out of data. Image flow cytometry was used to gain abundant single‐cell images, which were used for the screening system modeling, in a rapid and convenient way.

For evaluating the practicability of our model, popular methods for testing nanoformulated drugs were used as compared here. MTT assay is an absorbance‐based experiment which could measure the metabolic activity of living cells and be used frequently due to the advantage of easy to perform.^32^ It requires long experimental period since 24 h or longer treatment is usually needed for the testing.^33^ The whole experiment always needs 3–4 d, including seeding cells, proceeding treatment, coincubating with MTT, and reacting with dimethyl sulfoxide. While *DeepScreen* needs only minutes to judge the efficacy of different drugs with 2 h treatments, which means that the whole evaluation period could be reduced to several hours. Moreover, MTT assay has numerous other problems for the drug evaluation. First, as a colorimetric assay, MTT assay could be influenced by many factors, such as morphology and mitochondrial function of cells. MTT assay is not reliable for the drugs with an action on mitochondria.^34^ And, most importantly, MTT assay relates to cytostatic effect, neither of cytotoxicity, apoptosis nor viability of the treated cells.^35^, ^36^ Some cells could remain perfectly viable with low metabolic activity and it may cause “fake” data.^37^

Morphological approaches and directly cell apoptosis/necrosis measurements could avoid part of the limitation of MTT assay, but either of the above methods has its own technique problems: for crystal violet staining (CVS) assay, CV binds to all biomass, including microbes and slime which may cause background influence and staining itself could not discriminate between living and dead cells. As for flow cytometry, analysis distortion is difficult to avoid because of the side scatter background and complex offset setting.^38^ Moreover, they require similar or even longer period as MTT assay.

To build a reliable model, more interference in practical should be taken into consideration, some testing drugs like Cur could emit fluorescence and interfere with the absorbance values.^39^, ^40^ Besides, a considerable quantity of drugs need organic solvents to help dissolve; different organic solvents could have impact on MTT results.^41^

As for interference of nanomaterials, despite the reasons mentioned above such as the intrinsic high absorbance and optical activity, different morphology and components always bother researchers and every time for testing nanocarrier drug system, blank nanomaterial needs to be tested as well to avoid interference. But in this way, the interference cannot be actually eliminated, incubation time for nanocarrier and drug‐loaded system usually has own special morphology and properties, which could have totally different influence on measurement; independent test cannot reflect the impact of drug‐loaded nanocarrier.^25^, ^42^

Figure 4 indicates that despite the positive regulation on drug action against cancer cells of nanoparticles, MTT and flow cytometry analysis still fail to show differences of each treated strategies which have been already proved to have differentiated effect in 24 h MTT assay and previous studies.^7^, ^43^

High performance of the *DeepScreen* models suggests that our appropriately designed convolutional neural network can perform classification tasks using single‐cell IFC image data. One of the most important reasons why *DeepScreen* models can achieve this is that units in the convolution layers have similar information processing paradigm with human visual cortex. Studies suggested that human brain could piece together raw visual information into a complex object. Hubel and Wiesel discovered that parts of the cat's visual cortex are able to detect edges.^44^ Moreover, further work in neural science revealed that the visual cortex was organized in layers. In a convolution neural network, the lower layers are responsible for collecting primitive information about the image such as colors and dots.^45^ With the layers becoming higher, the collected information can be combined to form higher order information from lines, to contours, to basic shapes, to complex objects.^27^ Therefore, *DeepScreen* may identify the tiny localized variation of plasma membrane to perform classification tasks.

Moreover, we utilized class activation mapping technique to confirm our assumption. Figure 5 presents that the model targets specifically to the cell area in the input image for the correct class, whereas it may lose its focus or select meaningless random area with attention for the wrong classes. In addition, by comparison, it can be concluded that our model seems to be able to locate the tiny image area where the variation of the plasma membrane structure is proceeding. The fact that the additional Annexin V‐APC channel boosts the performance of our single bright‐field channel model by a large margin further proves the above assumption. Annexin V belongs to the annexin cellular protein group, which could conjugate to phosphatidylserine (PS). PS is located on the cytoplasmic surface of the cell membrane in normal condition and translocated from the inner to the outer leaflet of the cytomembrane.^46^ Based on this inference, more optimization can be applied by adding anti‐EGFR‐FITC channel to A549 cell model, resulting in an approximate error‐free detection. Epidermal growth factor receptor (EGFR) is a transmembrane glycoprotein which could regulate cellular proliferation and its expression reflects the progress of lung cancer.^47^

We also noticed that the interest of our model may concentrate on intracellular structures, which may indicate that *DeepScreen* has some ability to distinguish the regulation function of drug on cell cycle or proliferation. Another proof for the excellent location and identification abilities of our model is the high accuracy when dealing with nanocarrier drugs, through Figure S3 (Supporting Information) and Figure 1b, sometimes the nanoparticle‐treated cells will perform obvious morphologic changes, which made it hard to observe the cytomembrane as well as inner cell structures. As shown in Figure S3 (Supporting Information), our approach seems capable of ignoring the alienation parts and focus on the function areas.

The action of drug and nanoparticles on cell is not always focused on cell membrane and nuclei, other cellular components such as mitochondria and microtube are important target as well,^7^, ^33^ from Figure 5 and Figure S3 (Supporting Information), we also noticed that despite only cell membrane and nuclei were stained, *DeepScreen* focus not only contain these two parts, but also some other cellular content due to its powerful feature‐extraction ability. Since *DeepScreen* has the ability to extract the features by itself, possible upgrades for application could be expected to detect other cellular state changes such as stem cell differentiation. Related markers like Oct4, Sox2, etc., could be stained and detection by Flowsight,^48^ and the collected labeled images could be analyzed by our approach, a prediction system for stem cell differentiation could be built by training.

The increasing novel nanocarriers study requires the new screening method not only being able to analyze the common nanomaterials, but also the coming promising nanocarriers such as modularized extracellular vehicles (EVs).^49^, ^50^ EVs are nanosized cell‐derived vesicles which could be used to load small molecular drug, RNA, protein, etc., the growing attention of EVs poses a challenge to *DeepScreen*. EVs have cell membrane structure and when function as the drug carrier, some staining method may cause reaction of EVs membrane and it may lead to additional interference. Since our approach has strong anti‐interference on fluorescent drug, the distraction of EVs reflecting on images may have resemblance with fluorescent drug staining, *DeepScreen* may show similar superiority. The outstanding performance on evaluating both inorganic and liposome nanocarriers made us to believe that *DeepScreen* has the ability to deal with the drug‐loaded EVs nanocarrier efficacy evaluation; for further confirmation, certain number of labeled images of EV‐treated cells could be gained and used to upgrade *DeepScreen*.

Above findings make us believe that we could advance the application of *DeepScreen* system to more widen areas, not only for laboratory drug and nanocarrier drug screening, but also for in vivo or even clinic medication properties detection and evaluation, and it has the potential to upgrade for more demands.

## 4 Conclusions

In this study, we introduce a novel screen system for nanoformulated drugs, namely, *DeepScreen*. This screen system is based on deep learning which could be able to eliminate the human influences and the results show it is better suitable for detecting nanoformulated medication than existing laboratory approaches. Study shows that the novel method has outstanding performance as precise, fast, and convenient, it could contribute to research on the medical application of nanomaterials.

## 5 Experimental Section

*Data Preparation and Experimental Assays*: A549 cells (human pulmonary adenocarcinoma cell) and HEpG2 cells (human hepatocellular carcinoma cell) were usually used in in vitro models for the study of non‐small cell lung cancer and liver cancer, separately.^51^, ^52^ A549 and HEpG2 cell lines were purchased from the Chinese Academy of Sciences and maintained in RPMI‐1640/EMEM medium with10% fetal bovine serum (FBS) and 1% penicillin‐streptomycin (Gibco, BRL, Grand Island, NY).

Etoposide (VP16) and Cur were both known as efficient anticancer medications. LDH and SLN were widely used as nanocarriers in recent studies. LDH‐VP16 and SLN‐Cur were prepared with the established protocols as described.^7^, ^8^ TEM characterizations of LDH and SLN were established by transmission electron microscope (JEOL, Tokyo, Japan).

Cell cytotoxicity was measured using classical MTT assay following the established protocol.^7^ The improved methods were established to reduce the interference of drug fluorescence and nanoparticles. The half maximal inhibitory concentration (IC_50_) of each drug and nanocarrier drug system was obtained by calculating. The classification of drug efficacy was according to the MTT results.

HEpG2 and A549 cells were cocultured with different drugs/nanocarrier drug systems for 2 or 6 h, and then stained by Annexin‐V‐APC. A549 cells were stained by Anti‐ECFR‐FITC as well. For flow cytometry assay, cells were collected and analyzed by FACS Caliber (BD Bioscience). For gaining the training data of *DeepScreen*, cells were collected and photographed by Flowsight image flow cytometry (Amnis, Seattle, WA).

*Cell Image Data Processing*: Each cell image sample contains three single‐channel images. In order to generate the suitable inputs for the models, first, the single‐channel images of each cell image samples were concatenated channelwise. After that, these channelwise concatenated images were resized to images with height and width both being 70 pixels using the bicubic interpolation algorithm. Finally, the resized image samples were standardized to form the inputs for our models.

*Deep Learning Techniques: Convolutional Neural Network for Classification*: Based on the “Google Inception” network, a convolutional neural network was implemented to perform cell image recognition.^53^

Unlike common convolutional neural networks which usually have a straight layer‐by‐layer structure and end with multiple dense layers, the models adopt a “network‐in‐network” structure and replace dense layers with convolution layers.^54^ “Network‐in‐network” structure means that there are branches between two certain layers. Specifically, each of the branches can perform different convolution or pooling operation so long as they end with the same width and height and can be concatenated channelwise. This structure has already been proven to be effective in various computer vision tasks.^53^ In order to reduce the number of training parameters, none of the layers are densely connected, which means that there are only three types of layers in model, namely, convolution layer, pooling layer, and regularization layer like dropout layer.^55^ This kind of network configured ration can prevent overfitting to a large extent, but still has sufficient capability to extract high‐order features from input images.

*Model Training Methodology*: During the training process of the model, many techniques were applied which are widely used within the deep learning community. First, balanced sampling was adopted to form the mini‐batch for training, which means that there are equal numbers of samples from each class in every mini‐batch. This experiment shows that this sampling strategy has 1–2% performance improvement. Second, various regularization strategies were applied, namely, weight decay, dropout, and batch normalization.^56^ Utilization of these methods facilitates the generality of the model. Third, Adam optimizer,^57^ which combines the momentum and exponentially weighted moving average gradients methods, was used to update the training parameters within the network.

The model was trained utilizing the Tensorflow framework on two NVIDIA GTX 1080Ti GPUs. The parameters of Adam optimizer are as follows: learning rate of 0.001, beta1 of 0.9, beta2 of 0.999, and epsilon of 1 × 10^−8^.

*Class Activation Mapping for Feature Identification*: To gain a comprehensive view for understanding how the models recognize a cell image, the class activation mapping method was adopted.^29^ In order to generate class activation map, the last three layers in the model are follows: feature convolution layer, global average pooling layer, and 1 × 1 convolution layer without rectified linear units. Among the three layers, the feature convolution layer is simply a common convolution layer. With this structure, class activation map can be obtained with ease.

First, the weights of the final 1 × 1 convolution layer were extracted. After that, the forward pass was performed using a batch of input images and the feature maps of feature convolution layer were collected. Finally, the linear combinations of these feature maps and the weights were calculated to be the class activation map.

*Data Availability*: Code for the *DeepScreen* is available at https://github.com/yanjingmao/DeepScreen.git.

## Conflict of Interest

The authors declare no conflict of interest.

## Supporting information

Supplementary
