# Supplementary material for: DeepScreen: An Accurate, Rapid, and Anti‐Interference Screening Approach for Nanoformulated Medication by Deep Learning
Source: Adv Sci (Weinh). 2018 Jul 23;5(9):1800909. doi: 10.1002/advs.201800909 (PMC6145411; doi:10.1002/advs.201800909)
Supplement: Supplementary file 1 — Supplementary [file ADVS-5-1800909-s001.pdf]

Copyright WILEY-VCH Verlag GmbH & Co. KGaA, 69469 Weinheim, Germany, 2018.

ADVANCED SCIENCE
Open Access

Supporting Information

for *Adv. Sci.*, DOI: 10.1002/advs.201800909

DeepScreen: An Accurate, Rapid, and Anti-Interference Screening Approach for Nanoformulated Medication by Deep Learning

Yanjing Zhu, Ruiqi Huang, Rui Zhu, Wei Xu, Rongrong Zhu, and Liming Cheng**

SUPPORTING INFORMATION

1. EXPERIMENTAL SECTION：

1.1 Dynamic light scattering assay

Size distribution by intensity is determined using dynamic light scattering (DLS) at 25 °C by photon correlation spectroscopy (Zetasizer Nano ZS, Malvern Instruments, Malvern, UK), the LDH, LDH-VP16, SLN and SLN-Cur (1 mg mL−1) are prepared following the dilution that 100 μL samples diluted in 1 mL aqueous solution.

1.2 Fourier Transform Infrared Spectroscopy (FTIR) Spectral

FTIR of VP16, LDH, LDH-VP16, Cur, SLN and SLN-Cur was obtained on a Bruker Vector 22 (Bruker Corporation, Billerica, MA, USA) spectrophotometer in the range of 500–4000 cm^{-1} using the standard KBr disk method (sample: KBr = 1:100).

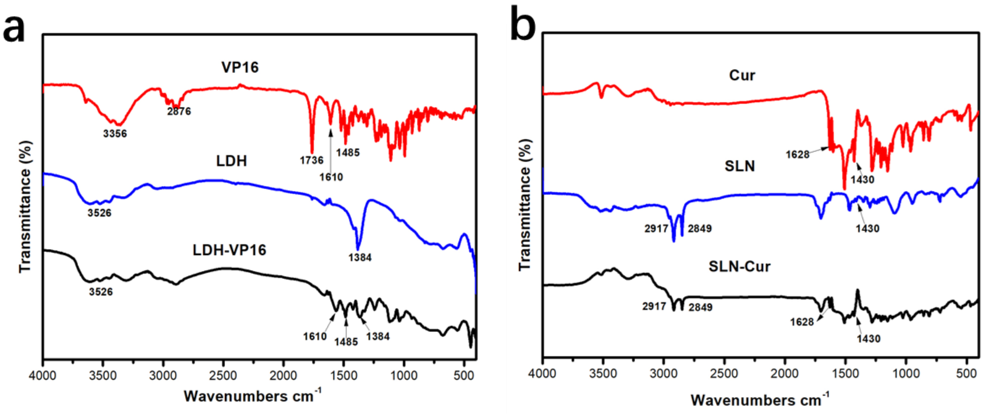

Figure. S1 FTIR spectra of **a)** VP16, LDH and LDH-VP16, **b)** Cur, SLN and SLN-Cur.

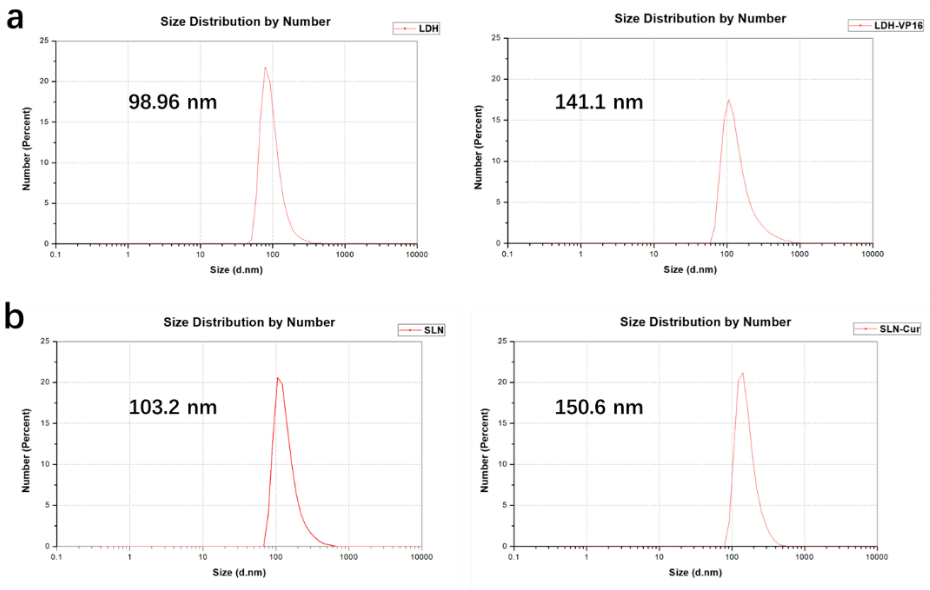

Figure. S2 Dynamic light scattering study of **a)** LDH and LDH-VP16, **b)** SLN and SLN-Cur.

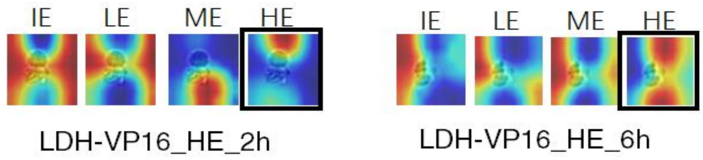

Figure. S3 The CAM highlights the class-specific discriminative regions of LDH-VP16 treated cells. IE, LE, ME and HE present ineffective, low, medium and high efficacy respectively.

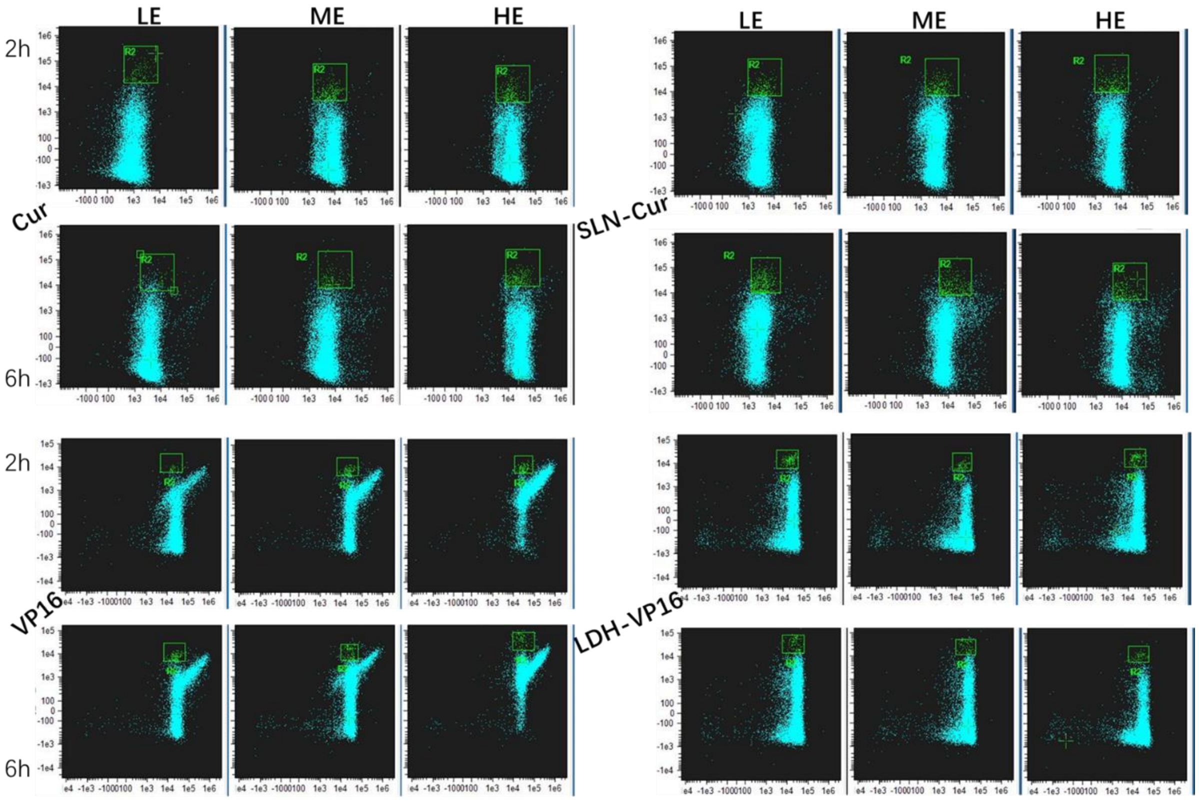

Figure. S4. The Flowsight cytometry results present no significant differenece among differnet dose or treated time in certain treated agent. *IE*, *LE*, *ME* and *HE* present ineffective, low, medium and high efficacy respectively.

Model	Test Data	Accuracy
A549-KRG	A549-KRG	0.966
A549-KR	A549-KR	0.909
A549-K	A549-K	0.714
HEpG2-KRC	HEpG2-KRC	0.888
HEpG2-KR	HEpG2-KR	0.835
HEpG2-K	HEpG2-K	0.669
A549+HEpG2-KR	A549+HEpG2-KR	0.851
	HEpG2-KR	0.864
	A549-KR	0.908
A549+HEpG2-K	A549+HEpG2-K	0.705
	HEpG2-K	0.667
	A549-K	0.770

Table S1. Accuracy value of different test set in each models, *K*, *R*, *G* and *C* present Bright-field, Annexin V-APC, anti-EGFR-FITC and self-fluoresce of Cur channel, separately.

exps	Corrects	Total	Accuracy
Ctrl-IE	1805	2257	0.799734
LDH-VP16_LE_2h	1313	1347	0.974759
LDH-VP16_LE_6h	1989	2201	0.90368
LDH-VP16_ME_2h	952	1035	0.919807
LDH-VP16_ME_6h	1284	1651	0.77771
LDH-VP16_HE_2h	1052	1115	0.943498
LDH-VP16_HE_6h	1419	1455	0.975258
VP16_LE_2h	2286	2508	0.911483
VP16_LE_6h	2233	2598	0.859507
VP16_ME_2h	2178	2359	0.923273
VP16_ME_6h	2257	2343	0.963295
VP16_HE_2h	2130	2391	0.890841
VP16_HE_6h	2622	2638	0.993935

exps	Corrects	Total	Accuracy
Ctrl-IE	1331	2015	0.660546
SLN-Cur_LE_2h	1384	1744	0.793578
SLN-Cur_LE_6h	1853	2296	0.807056
SLN-Cur_ME_2h	649	1878	0.34558
SLN-Cur_ME_6h	2601	2645	0.983365
SLN-Cur_HE_2h	1284	2203	0.582842
SLN-Cur_HE_6h	1507	1978	0.761881
Cur_LE_2h	1986	2049	0.969253
Cur_LE_6h	1993	2323	0.857942
Cur_ME_2h	1918	2333	0.822117
Cur_ME_6h	2547	2609	0.976236
Cur_HE_2h	2549	2635	0.967362
Cur_HE_6h	1569	2287	0.686052

Table S2. Accuracy of each treated groups in A549-HEpG2-Brightfeild&Annexin V-APC model. Up: accuracy of LDH-VP16/VP16 treated A549 cells test sets and down: accuracy of SLN-Cur/Cur treated HEpG2 cells test sets. *IE, LE, ME* and *HE* present ineffective, low, medium and high efficacy respectively.

exps	Corrects	Total	Accuracy
Ctrl-IE	2145	2257	0.950377
LDH-VP16_LE_2h	1339	1347	0.994061
LDH-VP16_LE_6h	2195	2201	0.997274
LDH-VP16_ME_2h	1027	1035	0.992271
LDH-VP16_ME_6h	1546	1651	0.936402
LDH-VP16_HE_2h	1040	1115	0.932735
LDH-VP16_HE_6h	1435	1455	0.986254
VP16_LE_2h	2478	2508	0.988038
VP16_LE_6h	2578	2598	0.992302
VP16_ME_2h	2267	2359	0.961
VP16_ME_6h	2335	2343	0.996586
VP16_HE_2h	2009	2391	0.840234
VP16_HE_6h	2627	2638	0.99583

Table S3. Accuracy of each treated groups in A549-Brightfeild&Annexin V-APC&anti-EGFR-FITC model. Test sets were collected from LDH-VP16/VP16 treated A549 cells. *IE, LE, ME* and *HE* present ineffective, low, medium and high efficacy respectively.

exps	Corrects	Total	Accuracy
Ctrl-IE	252	2257	0.111653
LDH-VP16_LE_2h	903	1347	0.670379
LDH-VP16_LE_6h	1960	2201	0.890504
LDH-VP16_ME_2h	975	1035	0.942029
LDH-VP16_ME_6h	1315	1651	0.796487
LDH-VP16_HE_2h	978	1115	0.87713
LDH-VP16_HE_6h	1072	1455	0.73677
VP16_LE_2h	2091	2508	0.833732
VP16_LE_6h	1986	2598	0.764434
VP16_ME_2h	2212	2359	0.937685
VP16_ME_6h	2057	2343	0.877934
VP16_HE_2h	1719	2391	0.718946
VP16_HE_6h	1756	2638	0.665656

exps	Corrects	Total	Accuracy
Ctrl-IE	606	2015	0.300744
SLN-Cur_LE_2h	601	2049	0.293314
SLN-Cur_LE_6h	2081	2323	0.895824
SLN-Cur_ME_2h	1431	2333	0.613373
SLN-Cur_ME_6h	2033	2609	0.779226
SLN-Cur_HE_2h	2346	2635	0.890323
SLN-Cur_HE_6h	1398	2287	0.611281
Cur_LE_2h	1355	1744	0.77695
Cur_LE_6h	1715	2296	0.746951
Cur_ME_2h	1395	1878	0.742812
Cur_ME_6h	1930	2645	0.729679
Cur_HE_2h	1027	2203	0.466182
Cur_HE_6h	1527	1978	0.771992

Table S4. Accuracy of each treated groups in A549-HEPG2-Brightfeild model. Up: accuracy of LDH-VP16/VP16 treated A549 cells test sets and down: accuracy of SLN-Cur/Cur treated HEpG2 cells test sets. *IE, LE, ME* and *HE* present ineffective, low, medium and high efficacy respectively.
